# Pharmacist intervention amid the coronavirus disease 2019 (COVID-19) pandemic: from direct patient care to telemedicine

**DOI:** 10.1186/s40545-020-00229-z

**Published:** 2020-05-27

**Authors:** Ali Elbeddini, Aniko Yeats

**Affiliations:** 1 Winchester District Memorial Hospital, 566 Louise Street, Winchester, ON KK0C2K0 Canada; 2 Leslie Dan Faculty of Pharmacy, University of Toronto, 144 college st, Toronto, M5S 3M2 Canada

**Keywords:** Telehealth, Geriatrics, Deprescribing, COVID-19

## Abstract

The coronavirus disease (COVID-19) pandemic has placed enormous pressures on the Canadian healthcare system. Patients are expected to stay home in order to contain the spread of the virus, but understandably have numerous questions and concerns about their health. With physical distancing being of utmost importance during the pandemic, much of healthcare has been forced to move online or over the telephone. Virtual healthcare, in the form of video calls, email, or telephone calls with patients, can significantly enhance access to healthcare. Many clinics have moved their appointments online, and physicians are seeing their patients by means of online video calls. Similarly, patients are refilling their prescriptions online and calling pharmacists whenever they have questions about their medications or medical conditions. Pharmacists are considered the most accessible primary care providers, so it is crucial for patients to know that pharmacists are there to support them throughout the pandemic.

## Discussion

### Impact of COVID-19 on the delivery of direct patient care

**Fig. 1:**
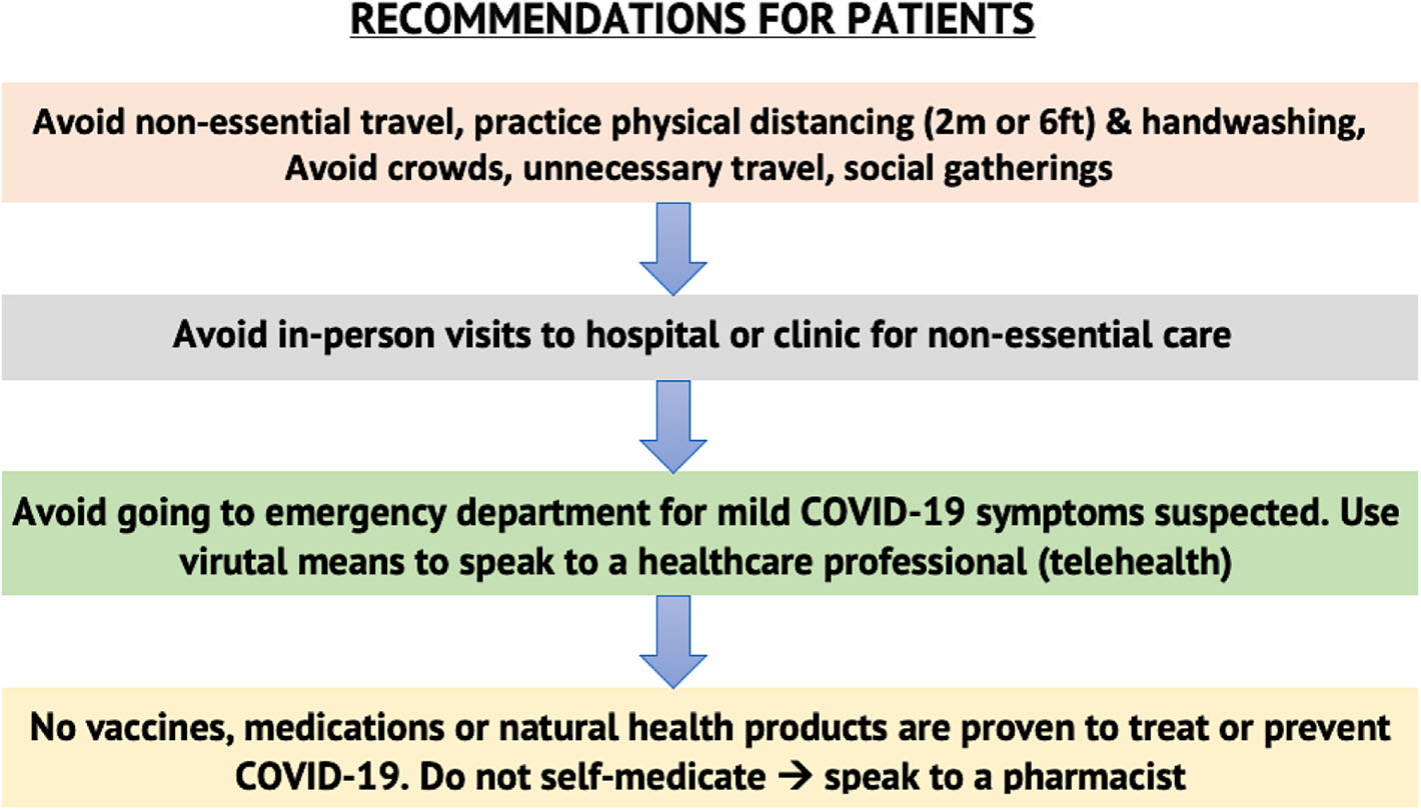
Recommendations for patients to help limit to spread of COVID-19. Following these steps will enable efficient use of healthcare resources [2]. Source: “Choosing Wisely COVID-19 Recommendations”

**Fig. 2:**
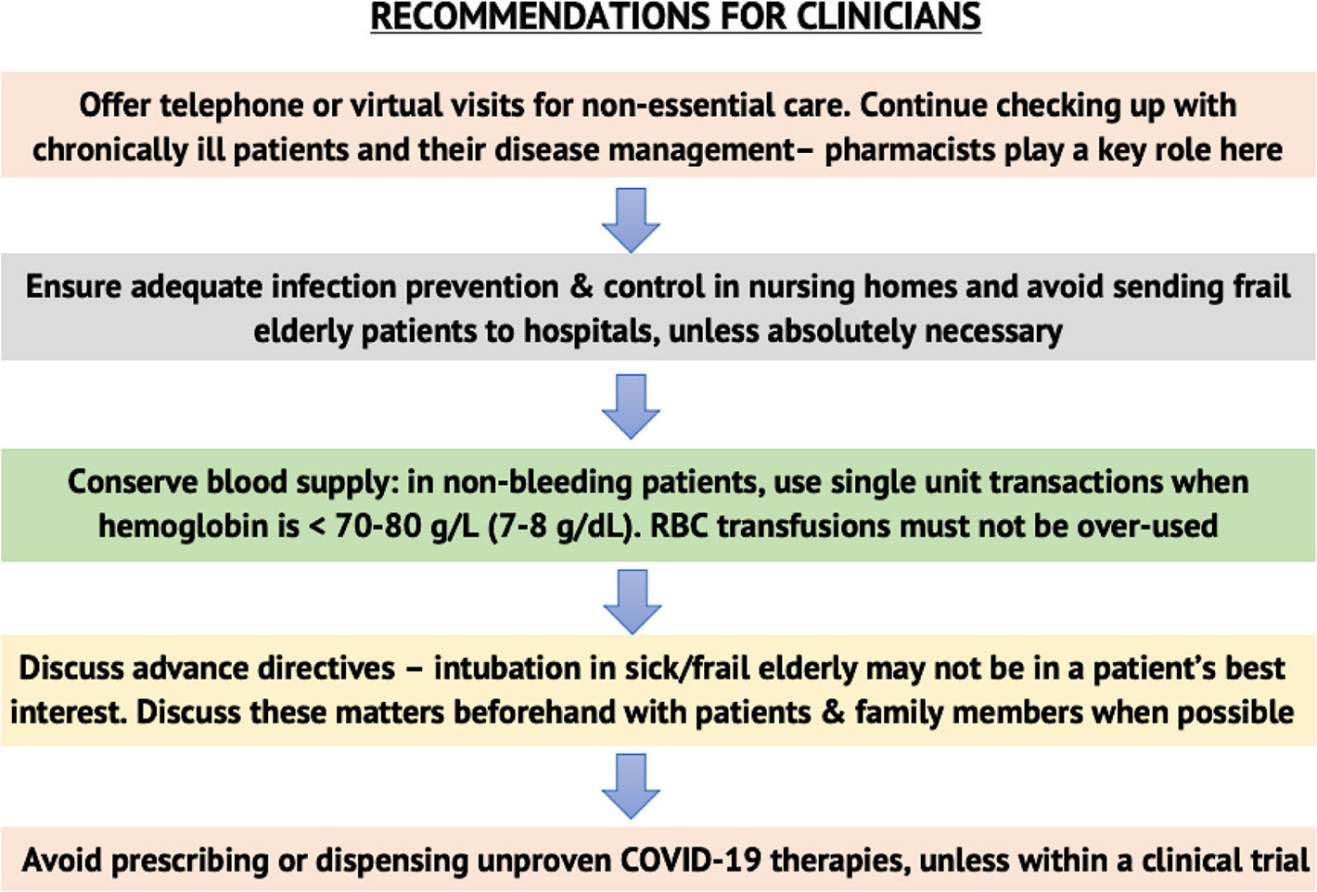
Recommendations for clinicians to help limit to spread of COVID-19. Following these steps will enable efficient use of healthcare resources [2]. Source: “Choosing Wisely COVID-19 Recommendations”

Hospital resources during the COVID-19 pandemic are limited, so we must ensure that patients are receiving the help they need while at home. Proper adherence to medications and management of chronic illness can greatly reduce the number of critical patients admitted to the hospital due to COVID-19. Uncontrolled blood glucose can result in microvascular and macrovascular complication of diabetes, which could compromise a patient’s survival if they contracted COVID-19 [1]. By means of telehealth, patients can be educated on their chronic conditions and medications over the phone, leading to a decreased need to physically show up to the pharmacy or clinic. The “Choosing Wisely COVID-19 Recommendations” are important for clinicians to keep in mind when counselling patients over the phone or in person (Figs. 1 and 2).

### Telemedicine as a means to optimize medication therapy

Telehealth has been shown to be a resource-effective way to deliver pharmacist services and to enhance patient care [3]. Patients in remote areas are able to receive the care they need, without having to physically come into the pharmacy. Various municipalities in Canada are discouraging non-essential travel during the COVID-19 pandemic, and this forced a large portion of health care to move online. Fortunately, many pharmacy services can be delivered over the phone, but the question arises about whether the quality of care by virtual means is on par with that of direct patient care. Many people also wonder whether it might be beneficial to continue with virtual healthcare after the pandemic is over, as it has proven to be a convenient and efficient way to provide healthcare services. Telemedicine is a current reality, and many healthcare providers, including pharmacists, play an important role in delivering this service.

### Managing multi-comorbid patients amidst COVID-19 through telemedicine

It has been shown that in patients with diabetes mellitus and hypertension, pharmacists are able improve adherence rates by means of telemedicine [4]. When glycemic control was looked at in patients with diabetes, it was found that telemedicine was about as effective as the standard of care [5]. Pharmacists are medication experts that are trained in determining actual and potential drug therapy problems. Having patients open up and trust pharmacists is important in order to get a complete and accurate medical history over the phone. Providing proper education and reassurance to patients can also reduce stockpiling of medications, a practice that may have devastating consequences. The Canadian Pharmacist Association (CPhA) has advised against stockpiling as it could exacerbate the current drug shortage problem and result in some patients going without essential medication. Pharmacies have limited their medications to a 30-day supply in order to avoid drug shortages, however this may cause a significant amount of stress on patients.

A randomized controlled trial looked at a telemedicine consultation device known as “GeriMedRisk” in the long-term care setting, and concluded that it feasible intervention to deliver virtual healthcare services to geriatric patients [6]. Elderly patients (≥ 65 years of age) often have an increased rate of adverse drug events due to multiple comorbidities, polypharmacy, and drug metabolism. “GeriMedRisk” is a telehealth platform used by pharmacists and physicians to help decrease adverse events in the elderly, namely falls and drug-related hospital visits [6]. One concern about telehealth is that the quality of care may not be as good as direct patient care. However, Suksomboon N et al. demonstrate that this is not the case, and that the quality of telemedicine can be as at least as good as direct patient care [5].

### The uncertainty around COVID-19 is as bad as the disease

Healthcare is moving to virtual means very quickly during this pandemic, so it is crucial that patients and clinicians both understand the importance of protecting privacy and confidentiality. This can be done by properly encrypting personal health information that comes in via email, and by asking for patient consent before beginning the online or telephone visits. Another important aspect of patient health that cannot be ignored is mental health, since it affects thousands of patients. Many people, especially those with dementia and multiple comorbidities, may feel afraid and uncertain about being left alone and having no one to care for them. Amid COVID-19, there has been a surge in mental health concerns among patients [7], so there must be a platform where patients can comfortably turn for help. Telemedicine can greatly improve access to professionals who can help patients suffering from mental illness. Interprofessional healthcare teams have already been implemented in Ontario offering telehealth to remote locations. With the COVID-19 pandemic, telehealth could evolve in such a way where virtual visits frequently include interprofessional teams (physicians, pharmacists, nurses, physiotherapists etc.), rather than just one clinician. This could drastically improve the access to and quality of patient care during the COVID-19 pandemic, but also moving forward.

### Deprescribing during the COVID-19 pandemic

Inappropriate drug therapy is costly not only to the patient but also financially to the healthcare system. An estimated $13.6 million is spent on treating adverse drug reactions in hospitals in Ontario [8] and in North America, approximately $419 million is spend on inappropriate drug therapy [6]. While many pharmacists currently use deprescribing as a means to optimize their patients’ health, it is not clear whether this practice is ideal during the pandemic. Research into COVID-19 is still underway and it is unknown which medications may be helpful or detrimental in a COVID-19 patient. The current recommendation is to hold back on deprescribing practices until the after the pandemic [9].

Deprescribing is a practice that has been shown to improve the lives of many older adults by preventing the rates of adverse drugs reactions, and reducing mortality, morbidity and hospital visits [10]. The practice of deprescribing could be safety done over the phone with some agents, but frequent monitoring would be necessary. This is something that could be looked into as the pandemic unfolds, as it has the potential to significantly reduce the incidence of ADR in elderly patients. Therefore, fewer elderly patients showing up to the emergency department due to reasons such as falls or drug toxicity.

## Conclusion

Telemedicine, or telehealth, is a convenient and cost-effective way to deliver healthcare to patients who reside in remote locations [6]. Many healthcare providers, including pharmacists, play an important role in delivering this service to patients. Ontario has had telehealth available since 2001 and many studies have shown that this service can positively impact patient care. Amid the COVID-19 pandemic, the demand for telehealth services has increased significantly, and governments are struggling to keep up. Patients are concerned about their health and are constantly looking for information regarding COVID-19. Over the phone, pharmacists can help patients recognize the signs of COVID-19 infection, guide them on how to manage their symptoms, and clarify any information regarding COVID-19. Furthermore, telehealth also allows pharmacists to monitor their patients’ chronic illnesses and to optimize their medications. Telemedicine has never been so widely used, and it leaves people wondering whether it might be beneficial to continue with virtual healthcare after the COVID-19 pandemic is over.

## Data Availability

Data sharing is not applicable to this article, as no datasets were generated or analyzed during the current study.
